# Development and initial validation of the Communication Inventory Disability – Observer Reported (CID-OR): a measure of communication in CDKL5 deficiency disorder

**DOI:** 10.1186/s41687-025-00977-z

**Published:** 2025-12-09

**Authors:** Jenny Downs, Jessica Keeley, Peter Jacoby, Sofia Benson-Goldberg, Sarah Pillar, Andrea Miele, Helen Leonard, Jacinta Saldaris, Eric D. Marsh, Tim A. Benke, Scott T. Demarest

**Affiliations:** 1 The Kids Research Institute Australia, University of Western Australia, Perth, WA Australia; 2 Curtin School of Allied Health, Faculty of Health Sciences, Curtin University, Perth, WA Australia; 3 Special Education and Clinical Sciences Department, College of Education, University of Oregon, Eugene, OR USA; 4 Department of Pediatrics and Neurology, University of Colorado School of Medicine, Precision Medicine Institute, Children’s Hospital Colorado, Aurora, CO USA; 5 Division of Neurology, Children’s Hospital of Philadelphia, School of Medicine, University of Pennsylvania, Philadelphia, PA USA; 6 Department of Pediatrics and Neurology, University of Colorado School of Medicine, Children’s Hospital Colorado, Aurora, CO USA

**Keywords:** CDKL5 deficiency disorder, Developmental and epileptic encephalopathy, Communication, Outcome measure, Psychometric

## Abstract

**Background:**

CDKL5 Deficiency Disorder (CDD) is a rare neurodevelopmental disorder characterised by early onset seizures combined with complex healthcare needs and developmental impairment that influence functional domains including communication. Communication is a high priority domain for families but currently used measures demonstrate floor effects. Emerging disease-modifying trials necessitate the precise and granular measurement of communication to determine drug efficacy. This study evaluated a new measure of communication for CDD called the Communication Inventory Disability – Observer Reported (CID-OR).

**Methods:**

CID-OR was developed using a communication framework for CDD created from qualitative data and informed through evaluation of current measures and consultation with clinical experts. Scores for items describing communicative purpose were derived from ratings of consistency and the mode of delivery. An online survey was administered to parents of people with CDD (*n* = 184) recruited from the International CDKL5 Clinical Research Network. Most caregivers (96%) completed the Communication and Symbolic Behavior Scales - Developmental Profile Infant Toddler Checklist (CSBS–DP ITC). A confirmatory factor analysis was conducted. Test-retest reliability and comparison of known groups were assessed using intraclass correlation coefficients and linear regression, respectively.

**Results:**

The median age of people with CDD was 9.8 (1–43) years. A single factor model was evaluated. For 34 items, item loadings were satisfactory. For the scale, internal consistency and goodness-of-fit statistics were satisfactory, except for the Root Mean Square Error of Approximation statistic. The distribution of CID-OR scores was less right skewed than for CSBS-DP ITC scores although there was a high correlation between CID-OR and CSBS scores (*r* = 0.89). Test-retest reliability was high (ICC 0.966).

**Conclusion:**

Preliminary analysis suggests that CID-OR is a reliable and valid measure of communication in CDD. This new tool has strong potential to measure responsiveness to interventions in CDD and may extend to similar developmental and epileptic encephalopathy conditions.

**Supplementary Information:**

The online version contains supplementary material available at 10.1186/s41687-025-00977-z.

## Background

CDKL5 deficiency disorder (CDD) is a rare Developmental and Epileptic Encephalopathy (DEE) caused by a genetic variant in *CDKL5* [1]. Symptoms include early onset epilepsy, global developmental delay, and other impacts on functioning and health such as cortical visual impairment, poor sleep, and gastrointestinal disturbances [1]. Individuals require daily care to support activities of daily living and complex health needs.

Each of the core functional domains of motor and cognitive skills, communication, and feeding are severely impacted. For example, approximately 33% are unable to sit, 75% unable to walk, and approximately 80% cannot use spoken words [2]. Approximately 25% are fed enterally [3]. In addition to achieving better seizure control, caregivers prioritize treatments that will address these developmental impairments, as even small improvements are anticipated to greatly improve quality of life for both individuals with CDD and their caregivers [4].

Communication has been expressed as a specific priority domain for families of people with CDD [4, 5]. However, the ability to directly measure the effectiveness of interventions on communication is limited by the challenges of accurate assessment in individuals with significant levels of intellectual disability [6]. Parent-report and direct clinical assessments of communication in very young children with developmental disorders or who were typically developing have been found to be generally consistent [7]. Valid parent-report measures of communication that are appropriate for the measurement of early communication skills in individuals with significant disabilities will have essential roles in documenting communication. Such measures can be used to evaluate the impacts of interventions and inform clinical decisions.

In a systematic review, 16 parent-report communication measures used in rare disease observational studies and clinical trials were identified where the Vineland Adaptive Behavior Scale was most frequently used [8]. Incomplete validation data in rare disease patient groups were found for six of the measures [8], although the Observer Reported Communication Assessment (ORCA) has comprehensive validation data for Angelman syndrome [9] and Rett syndrome [10] where functional impairments are generally less severe than CDD. Due to its ability to measure very early communication skills [11], we previously evaluated the validity and reliability of the Communication and Symbolic Behavior Scales - Developmental Profile Infant Toddler Checklist (CSBS–DP ITC) for CDD [12]. There were already existing validation data for the CSBS in some DEE conditions [e.g., 13], as well as report of responsiveness to change in a clinical trial evaluating trofinetide for Rett syndrome [14]. In CDD (*n* = 150), we found that goodness of fit and internal consistency statistics were strong, scores varied as expected by biological sex [i.e., lower score for males without mosaicism who typically experience more severe impairments than females; 1] and other functional abilities, and test-retest reliability was good [15]. On the other hand, the distribution of scores was right skewed where 58% of items were rated as 0 and the proportion of items rated 0 increased to 75% in people who could not walk, use spoken words or were gastrostomy fed [15]. The floor effect suggested inadequate granularity to capture methods of communication that are used by people with severe impairments, and that the CSBS may not be sensitive to change over time.

We subsequently undertook a concept elicitation study with 23 parents who have a child with CDD to explore their child’s communication [16]. Parents identified multiple non-symbolic and symbolic communication modes that their child used to express meaning, including communication of preferences, emotions, and social engagement with others [16]. Despite severely impaired communication, the data still illustrated richness and complexity [16], revealing that the individual useds skills that are not measured comprehensively in other communication tools [12]. Based on this qualitative framework and prompted by review of the literature [8], we constructed a new measure of communication for people with severe functional impairments. This paper presents the development and initial validation of the Communication Inventory Disability – Observer Reported (CID-OR) measure for people with CDD.

## Methods

This study was approved by the Internal Review Board at University of Colorado COMIRB 19-2756, with reliance agreements at each clinical site, and University of Western Australia (RA/4/20/6198). The International CDKL5 Clinical Research Network (ICCRN) is registered with ClinicalTrials.gov (NCT0555837).

### Development

The concepts and content validity of CID-OR were established through rigorous qualitative processes, as we have done previously [17–19]. Full details of the content validity of for CID-OR is published separately [20] but is summarized below for context.

This research was guided the following key principles: (1) receptive and expressive communication skills are intertwined and can be observed simultaneously [16], (2) communication is an expression of purpose through modes to form the communicative act and can be intentional [or non-intentional when the partner interpreted it as meaningful; 21, 22, 23], (3) communication partners need to understand meaning communicated through multiple non-symbolic and symbolic modes [16], and (4) symbolic language can be conveyed using words, signs, and alternative and augmentative communication (AAC) methods [16].

We created a framework from categories within the qualitative data and illustrated by quotes [16]. These categories were reviewed against concepts in existing communication measures, including the CSBS-DP ITC [12], the Communication Matrix [24], and the Vineland Adaptive Behavior Scales 3rd Edition [Vineland-3, communication domain; 25]. A suite of items was developed based on our qualitative data [16] but consistent with items in other measures. Where a concept from our communication framework was inconsistent with items in existing measures, unique items (*n* = 13/35, 37%) were created.

When completing CID-OR, parents were asked to report observable behaviours from the past 30 days. Each item is accompanied by examples able to be observed in people with less or more communication impairment [20]. First, each item evaluates the *consistency* of communication for a specific purpose (not currently, hardly ever, sometimes, often, consistently). This five-point scale reflects narratives provided in the initial qualitative dataset [16] and is consistent with clinical observations. Second, each *mode* of communication for that purpose is reported, including 5 groups of non-symbolic modes (moving limbs, moving whole body, facial expressions, eye movements, and vocalisations), and 4 levels of symbolic communication (single words/signs, 2 words in combination, 3 or 4 words in combination, or multiple sentences expressed in either spoken words, sign language, or using an AAC device). See Table 1.

**Table 1:**
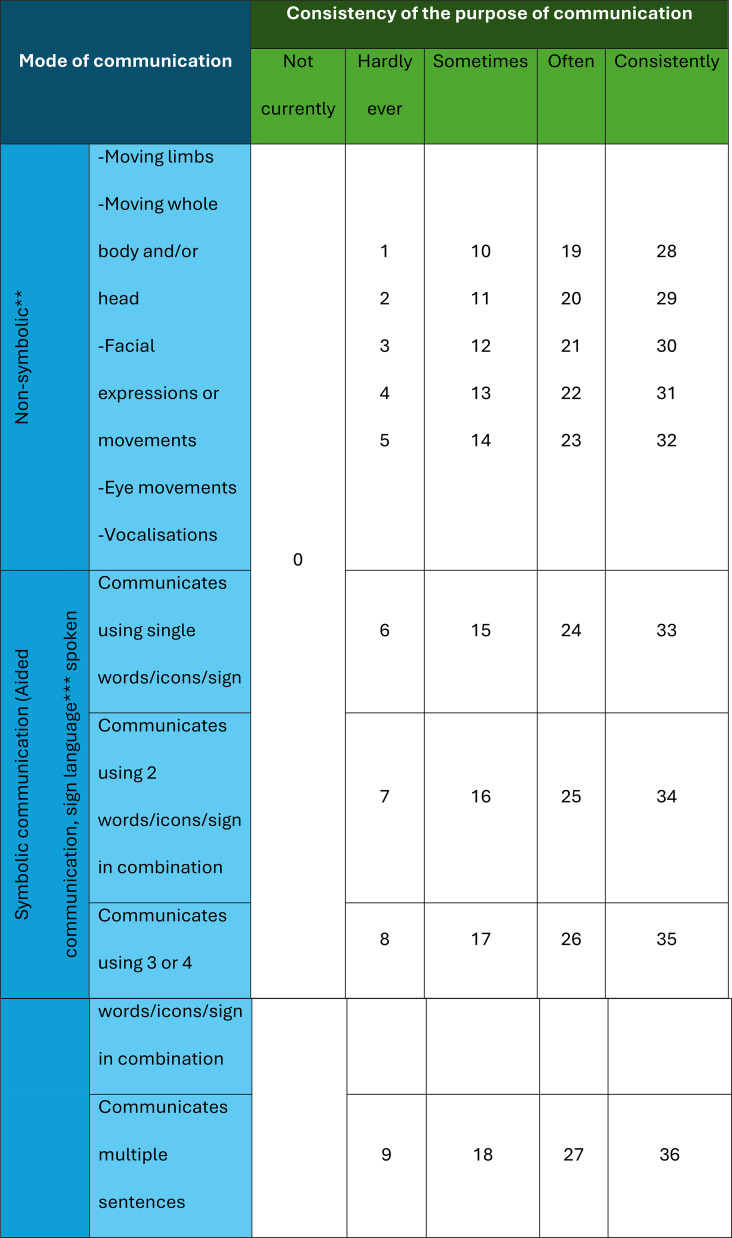
Table showing the bands of item scores for each level of consistency reported for each item*

* Individuals receive a score from a band of scores according to the consistency of the communication of the item. When the individual does not currently exhibit an item, they receive a score of zero. Individuals using non-symbolic communication modes only are given the sum of non-symbolic communication modes reported up to 5. Individuals who communicate symbolically are automatically awarded 5 points for non-symbolic communication with the item score determined by the complexity of symbolic communication. The maximum item score is 36 and the maximum scale score is 1224. The total score is then scaled to a score out of 100

** 1 point for each mode used and summed

*** Automatic 5 points for non-symbolic communication when symbolic communication used

The initial draft questionnaire comprised 35 items. Content validity was assessed by interviewing 21 parents/caregivers of children with CDD (17 females, median [range] age 15 [2–31] years) recruited from the International CDKL5 Disorder Database (ICCD) using ‘think aloud’ methodology to review clarity, comprehensiveness, and relevance of the items and measure [26, 27]. The measure was presented to six speech and language pathologists for feedback.

#### Scoring

Scoring combines observations on the consistency of the communication purpose and the mode of communication for each item, prioritising purpose over mode (Table 1). For each item, the response about consistency of purpose places the score for that item into a consistency band. Points are assigned according to the mode(s) of communication employed within each band. People with non-symbolic communication are given one point for each of the 5 non-symbolic modes employed. People who use symbolic communication are automatically credited with all non-symbolic communication skills and given a score ranging from 6 (e.g., using single words) to 9 (e.g., using multiple sentences) depending on use. The mode score is then added to the minimum score for each consistency band giving a total item score. The maximum possible item score is 36 for a person who consistently communicates a purpose using multiple sentences. Symbolic communication includes signs and use of an AAC device as well as spoken words. Equal weight is given to all three when scoring. Therefore, each combination of purpose and mode yields a unique score (Table 1). Seven allied health clinicians (speech and language pathology, neuropsychology) provided feedback on the clinical utility of the scoring system.

### Validation

#### Data sources

The ICCRN, comprising the CDD Centers of Excellence (CoE) network [28] and the ICDD [3] were the data sources for this study. Informed consent was obtained from all parent participants who provided data to the study.

Parents who attended a CoE in the USA since January 2023 were invited to participate in longitudinal clinic evaluations and completion of parent-reported questionnaires to describe their child’s comorbidities, communication, and quality of life. Assessments are repeated at approximately 6-month intervals for two years to track trajectories. An optional assessment is scheduled one month after the 12-month assessment to enable evaluation of test-retest reliability, completed by a subset of CoE participants. The CID-OR measure was introduced into the questionnaire battery in March 2024. The first completed CID-OR measure and test-retest reliability data are presented in this study. Families who were registered with the ICDD living internationally or in the US (but had not attended a CoE clinic) were invited to complete CID-OR between January and November 2024, if they spoke fluent English and had previously provided family questionnaire data to the database.

All people were required to have a CDKL5 variant that was considered pathogenic or likely pathogenic. Data were collected and managed using the Research Electronic Data Capture (REDCap) electronic data capture tool hosted at Children’s Hospital Colorado and The Kids Research Institute Australia.

#### Variables

In addition to CID-OR data, we collected data to describe person’s age, sex, and functional abilities. Walking ability was classified as walking without assistance, walking with assistance, or being unable to walk. Feeding ability was classified as oral only, some oral and some enteral feeding, or all enteral feeding. To assess convergent validity, some families completed the CSBS–DP ITC at the same time. The CSBS–DP ITC is a 24-item parent-report questionnaire that gives a total possible score of 57 [11, 12].

### Statistical analysis

The distribution of CID-OR scores and CSBS-DP ITC scores were compared visually in histograms, a scatterplot and using the skew statistic to assess any floor effects.

We applied Confirmatory Factor Analysis (CFA) to CID-OR responses using the *lavaan* package in R. The appropriate weighted least squares (WLS) estimator for items with ordinal levels was used. Factor loadings were calculated in addition to the following model fit statistics: Root Mean Square Error of Approximation (RMSEA), Standardized Root Mean Square Residual (SRMR), Comparative Fit Index (CFI), and Tucker-Lewis Index (TLI). These were compared with accepted thresholds [29]. Average Variance Extracted (AVE) was calculated. Discriminant validity of the multi-factor model domains was examined using the Fornell-Larcker criterion [30], i.e., by comparing AVE with the square of the maximum inter-domain correlation. Where necessary, the factor structure was re-organized, and items removed until satisfactory model statistics were achieved. Internal consistency was assessed using Cronbach’s Alpha.

The CID-OR questionnaire was completed on a second occasion after an interval of approximately 4 weeks, by a randomly selected subgroup (*n* = 26) of caregivers from the CoE group. Test-retest reliability for the total score was estimated using the Intraclass Correlation Coefficient (ICC). ICC values were interpreted as good (0.75–0.9) or excellent (> 0.9) reliability. Minimum Detectable Difference (MDD), that is the difference in score which can be said with confidence to be greater than measurement error, was calculated using the formula $\:MDD=z\surd\:2SD\surd\:(1-ICC)$ with values of $\:z=1.96$ for 95% confidence and $\:z=1.64$ for 90% confidence [31].   

We used linear regression to evaluate differences between known groups, to test the hypotheses that males without mosaicism, gastrostomy tube users, and those who could not walk would have lower communication scores than females, those able to feed orally and walk (with or without assistance), respectively.

Convergent validity was assessed using the Pearson correlation coefficient by comparing CID-OR scores with single factor scores from the CSBS-DP ITC.

## Results

There were 184 of 214 (86%) CID-OR questionnaires completed: 109 of 134 (81%) from the CoE dataset and 75 of 80 (94%) invited from the ICDD. There were no missing item data. The median (range) age for people with CDD was 9.8 (1–43) years. The majority were female (81.5%) as expected [32], 25% were able to walk independently and two thirds (63%) took all food orally. Characteristics of the participants and their communication scores are shown in Table 2.

**Table 2:** Characteristics of CID-OR questionnaire for all respondents and by data source

Participant characteristics	Median (IQR; min-max) / *n* (%)		
	All (*n* = 184)	COE (*N* = 109)	ICDD (*N* = 75)
**Age**,** years**	9.8 (5.1–17.1; 1.0-42.6)	8.7 (4.0-15.8; 1.0-42.6)	13.8 (6.8–18.2; 2.5–30.9)
**Age group**			
1–5 years	45 (24.5)	35 (32.1)	10 (13.3)
5–10 years	48 (26.1)	28 (25.7)	20 (26.7)
10–15 years	28 (15.2)	16 (14.7)	12 (16.0)
> 15 years	63 (34.2)	30 (27.5)	33 (44.0)
**Sex**,** n (%)**			
Female	150 (81.5)	91 (83.5)	59 (78.7)
Male*	34 (18.5)	18 (16.5)	16 (21.3)
**Ability to walk (*N*** **= 175)**,** n (%)**			
Walks without assistance	44 (25.1)	25 (25.0)	19 (25.3)
Walks with assistance	30 (17.1)	16 (16.0)	14 (18.7)
Unable to walk	101 (57.7)	59 (59.0)	42 (56.0)
**Feeding abilities**,** n (%)**			
Oral only	116 (63.0)	67 (61.5)	49 (65.3)
Some or all feeding enterally***	68 (37.0)	42 (38.5)	26 (34.7)
**CID-OR score (*N*** **= 184)**	22.5 (12.4–38.2; 0.1–95.6)	21.8 (11.3–41.5; 0.1–95.6)	23.4 (12.9–32.2; 0.2–88.2)
**CSBS score (*N*** = 176) **	17.5 (10.5–28.1; 0-100)	17.6 (10.5–31.6; 0-100)	15.8 (8.8–22.8; 1.8–91.2)

* Includes 10 males with identified mosaicism

** Maximum possible score of 57 scaled to maximum possible score of 100 for comparability with CID-OR scores

*** 27 in the COE cohort were fully fed enterally, 14 in the ICDD cohort were fully fed enterally

### Confirmatory factor analysis

Initially a 5-factor CFA model was examined based on initial conceptual grouping of items suggested in the qualitative study, consisting of the groups of items describing Demonstrating Preferences (6 items), Indicating Understanding (9 items), Expressing Emotions (9 items), Social Connections (6 items), and Two-Way Exchanges (5 items). Goodness of fit statistics were satisfactory, but the discriminant validity requirement was not satisfied due to very high inter-domain correlations. A single factor model was subsequently evaluated. This model performed well apart from a low factor loading (0.3) for one item (*specific communication used with specific people*). This item was removed resulting in the final single factor 34-item CID-OR questionnaire (Table 3).

**Table 3:** CID-OR item factor loadings (*n* = 34 items). Summary of item content is presented in the CID-OR item content column

CID-OR item	Factor loading	95% confidence interval
Does your child		
1. Indicate preference for people/activities	0.840	0.805–0.875
2. Indicate not wanting something	0.793	0.750–0.835
3. Indicate wanting more of something	0.835	0.796–0.874
4. Make choices between items	0.815	0.773–0.857
5. Indicate yes/no to a question	0.803	0.758–0.849
6. Let you know they need help	0.711	0.657–0.764
7. Respond to their name	0.727	0.673–0.780
8. Anticipate preferred activities	0.844	0.804–0.885
9. Persist when not understood	0.809	0.769–0.850
10. Follow one-step instructions	0.841	0.801–0.881
11. Follow two-step instructions	0.943	0.906–0.980
12. Recognise familiar objects/people	0.774	0.727–0.821
13. Recognise familiar objects spontaneously	0.779	0.730–0.829
14. Respond in expected way	0.830	0.777–0.883
15. Describe things using actions/gestures	0.906	0.859–0.953
16. Indicate when they are happy	0.830	0.792–0.867
17. Indicate that they are excited	0.826	0.786–0.866
18. Indicate that they are upset/sad	0.755	0.707–0.803
19. Indicate that they are worried/scared	0.833	0.786–0.881
20. Indicate that they are frustrated	0.732	0.679–0.785
21. Indicate that they are angry	0.736	0.677–0.795
22. Indicate that they are uncomfortable	0.612	0.552–0.673
23. Indicate that they are in pain	0.603	0.536–0.669
24. Indicate location of pain	0.792	0.735–0.850
25. Try to get your attention	0.841	0.803–0.879
26. Try to get you to notice objects	0.885	0.840–0.93
27. Show/accept affection	0.742	0.693–0.790
28. Do things to please you	0.873	0.826–0.921
29. Play with others	0.851	0.806–0.895
30. Greet you	0.788	0.735–0.841
31. Make or imitate noises	0.764	0.709–0.819
32. Respond to questions	0.879	0.833–0.925
33. Give you information	0.897	0.807–0.988
34. End an interaction	0.674	0.592–0.756

Fit statistics for the final model are shown in Table 4. The SRMR and TLI values were > 0.9 indicating good model fit. The RMSEA was higher than the recommended threshold. However, RMSEA is not recommended as a fit measure for use in models with a small sample size (*n* < 200) where the SRMR is considered a preferable indicator of model fit [33]. Average Variance Extracted (AVE) with the 34 items was 0.64 which is above the recommended minimum of 0.5. Cronbach’s alpha was 0.97 indicating excellent internal consistency of the scale.

**Table 4:** Fit statistics of the single factor CFA model with 34 items

Fit statistic	Observed	Recommended threshold*
Chi-square (degrees of freedom)	1338.9 (527)	
Chi-square / df	2.54	< 3
Root Mean Square Error of Approximation (RMSEA)	0.092	< 0.08
Comparative Fit Index (CFI)	0.97	> 0.9
Tucker-Lewis Index (TLI)	0.96	> 0.9
Standardised Root Mean Square Residual (SRMR)	0.065	< 0.08

* Hu L, Bentler PM. Cutoff criteria for fit indexes in covariance structure analysis: Conventional criteria versus new alternatives. *Structural Equation Modeling: A Multidisciplinary Journal* 1999;6:1–55

### Distribution of scores

**Fig. 1:**
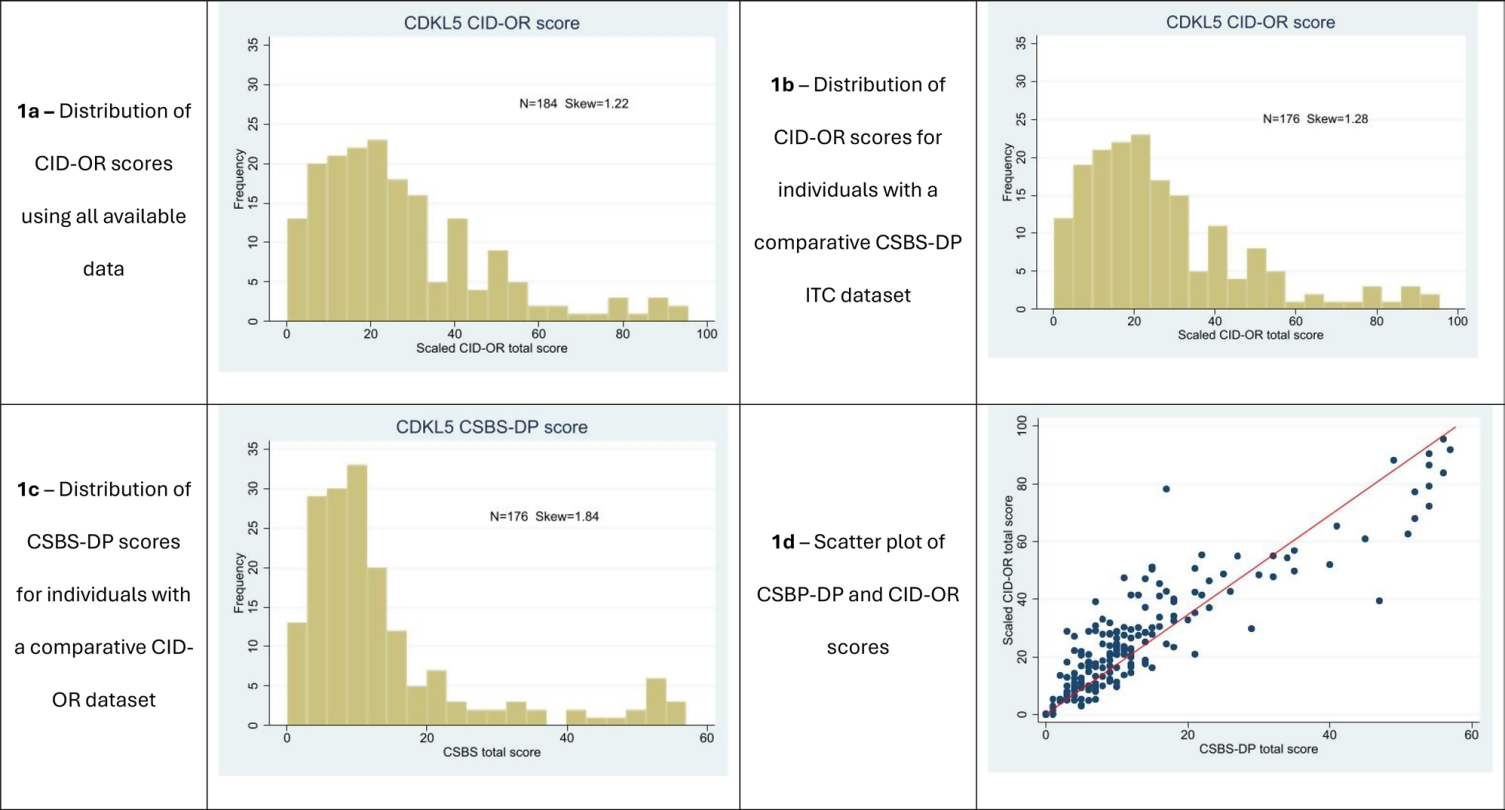
Histograms and scatterplot showing distributions of CID-OR and CSBS-DP scores

The median CID-OR score (*n* = 184) was 22.5 (skew 1.22, Fig. 1a), out of a total possible score of 100. No person scored 0 points. For the 176 participants who completed both communication questionnaires, the median CID-OR score was 22.2 (skew 1.28) and the median scaled CSBS-DP ITC score was 17.5 (skew 1.84). There was less right skew in the CID-OR score distribution than in the CSBS-DP score distribution (Fig. 1b, c). Three (1.7%) people scored 0 points on the CSBS-DP. The mean number of items scored as 0 was 45% for CID-OR compared with 63% for CSBS-DP. Items for the person indicating they are happy, showing affection, and indicating they did not want something had the highest median CID-OR item scores whilst items for following two-step instructions, describing things with actions or gestures, and giving information had the lowest median scores (Supplementary Table 1). Case studies derived from the dataset are presented to illustrate skills associated high and low scores, and variability by consistency of communication (Supplementary Table 2).

### Convergent validity

For the 176 people with CID-OR and CSBS-DP ITC data, scores were strongly positively correlated (*r* = 0.89). The scatter plot illustrates a greater range of CID-OR scores for low CSBS-DP ITC scores, suggesting that CID-OR reduced the floor effect of the CSBS-DP (Fig. 1d).

### Known groups validity

Results of the known-groups validity analysis are presented in Table 5. As hypothesized, females had a higher mean CID-OR score than males (after removal of males with reported mosaicism) although this effect did not reach statistical significance. People who fed orally had significantly higher scores than people who were reliant on a gastrostomy tube for feeding and people unable to walk had significantly lower scores than people who could walk independently or with assistance.

**Table 5:** Known groups comparison of CID-OR scores by feeding ability, mobility and sex categories

	Mean CID-OR score	95% CI	Effect size (95% CI) *p*-value
**Feeding**			
Gastrostomy tube (*N* = 68)	17.86	14.40–21.32	15.19 (9.38–21.01) *P* < 0.001
Oral feeding (*N* = 116)	33.06	29.07–37.04	
**Mobility (*N*** **= 175)**			
Unable to walk (*N* = 101)	18.20	14.94–21.46	Ref
Walks with assistance (*N* = 30)	27.93	21.95–33.91	9.73 (2.92–16.54) *P* = 0.005
No assistance needed (*N* = 44)	48.60	43.66–53.53	30.40 (24.48–36.31) *P* < 0.001
**Sex (*N*** **= 172)**			
Male (*N* = 22*)	21.02	24.04–30.33	6.17 (-2.53–14.87) *P* = 0.16
Female (*N* = 150)	27.19	29.07–37.04	

* 12 males with reported mosaicism removed

### Test-retest reliability and MDD

Test-retest reliability, calculated from repeat administration after a mean (SD) of 33.9 (7.1) days (*n* = 26 from the ICCRN dataset), was excellent with an ICC value of 0.966 (95%CI 0.925–0.984). The Minimum Detectable Difference (MDD) was calculated to be 10.53, corresponding to the CID-OR score change where there is 95% confidence that the change is greater than measurement error. MDD corresponding to 90% confidence was 8.81.

## Discussion

Communication is a high priority domain for parents of children with CDD [4, 5]. We developed CID-OR to measure communication in CDD because available data suggested floor effects in existing parent-report measures [15]. Thus, the observable and practical communication skills that people living with a severe disability learn and use in their everyday life were not being measured. The development process involved mapping concepts from a rich qualitative dataset [16] against concepts in existing measures [12, 24, 25], and generating new items. We then used ‘think aloud’ for content validation and consultation with clinicians to further inform the structure and presentation of CID-OR. Initial validation provided good evidence of goodness of fit for a single domain, known groups validation, and test-retest reliability. Importantly, there was reduced right skew for CID-OR scores compared to scores derived from the CSBS-DP ITC.

CID-OR uses a novel questionnaire structure to measure communication behaviours [21] for individuals with severe communication impairments. In sum, receptive and expressive communication skills are demonstrated within the one item set, consistency of purpose and mode of communication are rated for each item, and equal weight is afforded to different modes of symbolic communication. The scoring then prioritises *consistency of purpose over mode*, based on parent and clinician feedback to measure functional communication. Bands of scores are applied to each level of consistency with higher scores for more complex symbolic modes of communication within each band. Each combination of purpose and mode has a unique score. The scoring of CID-OR credits symbolic communicators with the non-symbolic modes of communication. Preliminary analysis identified that the symbolic communicators were sometimes receiving low mode scores that did not reflect their more complex communication because parents were not recording the more subtle forms of communication (i.e., non-symbolic communication modes such as facial expressions, eye movements). The automatic application of non-symbolic communication modes to the score was included after a thorough review of scores and in consultation with healthcare professionals with extensive experience working with individuals with CDD and other DEE conditions within and outside the team.

To our knowledge, the structure of the CID-OR questions and scoring is unique although there are some commonalities with other parent-report communication measures. For example, CID-OR generates a single score consistent with the communication domain of the Developmental Profile 4th Edition [34], and the Observer Reported Communication Assessment developed for Angelman syndrome [9] and adapted for Rett syndrome [10]. However, the Vineland-3 communication subscale has additional scales for expressive, receptive, and written communication [25]. There are some commonalities with the Communication Matrix [24] where the purpose is to generate a profile; first classifying by different modes of symbolic communication and then coding the purpose as emerging or mastered and generating a map across different modes. However, we are not aware of any communication measure that privileges consistency of purpose over mode nor generates combined purpose and mode item and total scores.

After establishing a model of communication in CDD [16] and consulting with healthcare professionals [35], we evaluated CID-OR’s structural characteristics, construct validity, and test-retest validity [36]. Testing the themes derived from the qualitative data as domains in a confirmatory factor analysis resulted in poor discriminant validity. Rather, the model fitted well for a single domain, potentially indicating that communication is a complex interactive skill at every level of ability and that multiple skills are being acquired simultaneously as communication develops. For example, individuals with greater communication impairments demonstrated components of each of the qualitative themes but with lesser consistency or using less symbolic modes.

Initial evidence for reliability and validity indicates many future potential studies including evaluation of responsiveness to change, the determinants of communication in CDD, and relationships with the person’s quality of life and parent burden. We utilized case studies to illustrate variability across the spectrum of scores for six individuals in the dataset (Supplementary Table 2), including for individuals with similar scores but different communication profiles. Further evaluation is needed to determine if CID-OR could be a helpful clinical tool for tracking the ways that a person is communicating and for supporting parents, caregivers and educators to attune sensitively to their child’s unique communication methods. For an ultrarare disorder [37], our sample size is substantial and the individuals with CDD in this study were broadly similar to those in other studies [1]. Nevertheless, a larger sample size would be necessary for us to evaluate model fit using the RMSEA statistic [33]. The Cronbach’s alpha value was high which could reflect the relatively large number of items and suggest some redundancy. However, the items each indicate different acts of communication and after randomly splitting the items into 2 groups, the Cronbach’s alpha values were 0.944 and 0.942.

## Conclusions

CDD is one of many DEE conditions with a known genetic cause [38] and the number of identified conditions will only increase with further advances in diagnostic technologies [38]. DEE conditions are defined phenotypically, and common features are well represented by CDD. Accordingly, we expect CID-OR to have broad applicability across multiple DEEs or other conditions with severe intellectual disability that share a similar phenotype. Establishing validation datasets of these similar disorders is an important next step. There is potential for the use of CID-OR in clinical trials.

## Supplementary Information

Below is the link to the electronic supplementary material.

Supplementary Material 1

## Data Availability

Data are available from the authors on reasonable request with appropriate ethical approvals.

## Abbreviations

AVE: Average Variance Extracted
CDD: CDKL5 deficiency disorder
CFA: Confirmatory Factor Analysis
CoE: Centers of Excellence
CFI: Comparative Fit Index
CID-OR: Communication Inventory Disability – Observer Reported
CSBS–DP ITC: Communication and Symbolic Behavior Scales - Developmental Profile Infant Toddler Checklist
DEE: Developmental and Epileptic Encephalopathy
ICCRN: International CDKL5 Clinical Research Network
ICDD: International CDKL5 Disorder Database
ICC: Intraclass Correlation Coefficient
MDD: Minimum Detectable Difference
ORCA: Observer Reported Communication Assessment
RMSEA: Root Mean Square Error of Approximation
SRMR: Standardized Root Mean Square Residual
TLI: Tucker-Lewis Index
