# Supplementary material for: Development and initial validation of the Communication Inventory Disability – Observer Reported (CID-OR): a measure of communication in CDKL5 deficiency disorder
Source: J Patient Rep Outcomes. 2025 Dec 9;10:6. doi: 10.1186/s41687-025-00977-z (PMC12799820; doi:10.1186/s41687-025-00977-z)
Supplement: Supplementary file 1 — Supplementary Material 1 [file 41687_2025_977_MOESM1_ESM.docx]

**Supplementary Table 1** – Median item score (IQR) and the number (%) scoring zero for each CID-OR item (N=184)

| **CID-OR item***  **Does your child……^** | **Median raw score** | **IQR** | **Number (%) scoring zero** |
| --- | --- | --- | --- |
| 1. Indicate preference for people/activities | 15 | 10.5 - 25 | 23 (12.5%) |
| 2. Indicate not wanting something | 21 | 12 - 30 | 11 (6.0%) |
| 3. Indicate wanting more of something | 12 | 0 - 24 | 49 (26.6%) |
| 4. Make choices between items | 10 | 0 - 19 | 70 (38.0%) |
| 5. Indicate yes/no to a question | 0 | 0 - 15 | 100 (54.3%) |
| 6. Let you know they need help | 11 | 0 - 22 | 50 (27.2%) |
| 7. Respond to their name | 11 | 2 - 20.5 | 31 (16.8%) |
| 8. Anticipate preferred activities | 10 | 0 - 20 | 69 (37.5%) |
| 9. Persist when not understood | 10 | 0 - 20 | 67 (36.4%) |
| 10. Follow one-step instructions | 2.5 | 0 - 15 | 79 (42.9%) |
| 11. Follow two-step instructions | 0 | 0 - 0 | 147 (79.9%) |
| 12. Recognise familiar objects/people | 10 | 0 - 21 | 72 (39.1%) |
| 13. Recognise familiar objects spontaneously | 0 | 0 - 13.5 | 102 (55.4%) |
| 14. Respond in expected way | 0 | 0 - 6.5 | 123 (66.8%) |
| 15. Describe things using actions/gestures | 0 | 0 - 0 | 162 (88.0%) |
| 16. Indicate when they are happy | 20 | 11 - 29.5 | 12 (6.5%) |
| 17. Indicate that they are excited | 13 | 0 - 23 | 50 (27.2%) |
| 18. Indicate that they are upset/sad | 12 | 1 - 23.5 | 44 (23.9%) |
| 19. Indicate that they are worried/scared | 0 | 0 - 12.5 | 104 (56.5%) |
| 20. Indicate that they are frustrated | 11 | 0 - 18.5 | 57 (31.0%) |
| 21. Indicate that they are angry | 0 | 0 - 15 | 94 (51.1%) |
| 22. Indicate that they are uncomfortable | 12 | 10 - 22 | 25 (13.6%) |
| 23. Indicate that they are in pain | 12 | 1 - 23 | 43 (23.4%) |
| 24. Indicate location of pain | 0 | 0 - 0 | 142 (77.2%) |
| 25. Try to get your attention | 12 | 0 - 22 | 52 (28.3%) |
| 26. Try to get you to notice objects | 0 | 0 - 0 | 144 (78.3%) |
| 27. Show/accept affection | 20.5 | 11 - 30 | 21 (11.4%) |
| 28. Do things to please you | 0 | 0 - 12 | 123 (66.8%) |
| 29. Play with others | 0 | 0 - 5.5 | 130 (70.6%) |
| 30. Greet you | 10.5 | 0 - 21 | 65 (35.3%) |
| 31. Make or imitate noises | 0 | 0 - 10 | 117 (63.6%) |
| 32. Respond to questions | 0 | 0 - 0 | 145 (78.8%) |
| 33. Give you information | 0 | 0 - 0 | 171 (92.9%) |
| 34. End an interaction | 0 | 0 - 15 | 102 (55.4%) |

* Maximum item score is 36, maximum total raw score is 1224, scaled to a 0–100-point scale.

^ Summary phrase for each item presented.

**Supplementary Table 2** – Case studies illustrating variation in consistency and modes of communication, presented in order of score magnitude, followed by summary vignettes that illustrate communication strengths for each individual.

| **Child** | | | | | **Scores** | | | | | |
| --- | --- | --- | --- | --- | --- | --- | --- | --- | --- | --- |
| Child | Age (years) | Sex | Walks on own | G-tube | Total raw / scaled score^ | Consistency  (n [%] of items) | | Mode  n items* | | Summary vignette |
| 1 | 5 | Female | No | No | 40 / 3.27 | Consistently  Often  Sometimes  Hardly ever  Not currently | 0  0  2 (6%)  11 (32%)  21 (62%) | Limbs  Whole body  Facial expression  Eyes  Vocalisations | 2  9  3  2  5 | Non-symbolic communication only. Primarily uses whole body movements to communicate preferences (wanted/not wanted), emotions (happy, excited, discomfort). Shows/accepts affection infrequently. Multiple modes used to express happiness. |
| 2 | 5 | Female | No | No | 166 / 13.56 | Consistently  Often  Sometimes  Hardly ever  Not currently | 2 (6%)  3 (9%)  3 (9%)  8 (23%)  18 (5%) | Limbs  Whole body  Facial expressions  Eyes  Vocalisations | 1  12  9  0  11 | Non-symbolic communication only. Primarily uses whole body movements and vocalisations to communicate. Consistently communicates to indicate when did not want or like something or if in pain. |
| 3 | 6.5 | Female | Yes | No | 338 / 27.61 | Consistently  Often  Sometimes  Hardly ever  Not currently | 0  7 (20%)  13(38%)  4 (11%)  10 (29%) | Limbs  Whole body  Facial expressions  Eyes  Vocalisations  AAC device  (Single icon) | 14  21  17  16  20  1 | Mix of symbolic and non-symbolic communication modes. No communication purpose was consistent. Uses multiple communication modes. Communicates non-symbolically often when feeling happy, excited and angry, and persists when not understood. Communicates humour. |
| 4 | 15 | Female | No | Yes | 480 / 39.22 | Consistently  Often  Sometimes  Hardly ever  Not currently | 12 (35%)  6 (18%)  0  0  16 (47%) | Limbs  Whole body  Facial expressions  Eyes  Vocalisations | 14  10  5  2  16 | Non-symbolic communication only. Consistently communicates emotions except when feeling pain and some preferences. Consistently ends an interaction. Uses a range of modes, predominantly vocalisations and limb movements. Communicates humour. |
| 5 | 6 | Female | No | No | 508/41.50 | Consistently  Often  Sometimes  Hardly ever  Not currently | 12 (35%)  3 (15%)  2 (6%)  5 (9%)  12 (29%) | Limbs  Whole body  Facial expressions  Eyes  Vocalisations | 18  18  17  3  20 | Non-symbolic communication only. Uses a range of modes (most often vocalisations) across a range of consistencies. Consistently expresses likes and dislikes, feelings of discomfort or pain, and acts as expected in social situations. |
| 6 | 11 | Male | Yes | No | 1,125 / 91.91 | Consistently  Often  Sometimes  Hardly ever  Not currently | 24 (71%)  9 (26%)  1(3%)  0  0 | Spoken words –Multiple sentences for all items | 34 | Mostly, communicated purposes consistently using symbolic and non-symbolic communication for multiple purposes. Advanced conventional communicator using multiple sentences. |

^The maximum item score is 36 and the maximum scale score is 1224. The total score is then scaled to a score out of 100.

*When symbolic communication is used for a purpose, non-symbolic is not presented as automatic non-symbolic scores are awarded.
